# Case Report: Serial changes of ocular complications related to immune checkpoint inhibitors pembrolizumab and nivolumab

**DOI:** 10.3389/fopht.2022.1021574

**Published:** 2023-01-05

**Authors:** Wan-Hua Cho, Wei-Yu Chiang

**Affiliations:** ^1^ Department of Ophthalmology, Chi Mei Medical Center, Tainan, Taiwan; ^2^ Department of Ophthalmology, Kaohsiung Chang Gung Memorial Hospital and Chang Gung University College of Medicine, Kaohsiung, Taiwan

**Keywords:** immune checkpoint inhibitors, pembrolizumab, nivolumab, ovarian cancer, uveitis, maculopathy

## Abstract

**Background:**

To demonstrate the rare ocular side effects in a patient receiving pembrolizumab and nivolumab for metastatic ovarian cancer.

**Case presentation:**

A 37-year-old woman with recurrent metastatic ovarian cancer presented with blurred vision and photophobia after receiving pembrolizumab. Ocular findings were bilateral anterior chamber reactions, iris cysts, and macular flecks. Optical coherence tomography (OCT) indicated retinal pigment epithelium (RPE) and ellipsoid-band disruption. Her symptoms subsided with topical steroids but fundal appearance persisted despite cessation of immunotherapies. Similar episodes attacked again with multiple exudative subretinal fluid (SRF) developed after she received pembrolizumab and nivolumab. Steroids could cease anterior chamber reactions while SRF only subsided after discontinuation of immunotherapy. Extensive RPE and ellipsoid-band disruption remained without vision improvements.

**Conclusions:**

We report a rare case of uveitis and retinopathy after immunotherapies with sequent pembrolizumab and nivolumab. A serial change of the maculopathy is demonstrated. Possible ocular toxicities during the treatment course should be considered, and the benefits of continuing the immunotherapy must be weighed against the risks.

## Background

Recently, immunotherapy has been applied for clinical use in cases of malignancies to treat tumor regression and prolong the survival (1). Immune checkpoint inhibitors are immunomodulatory antibodies used to enhance the immune system function. They have been utilized in cases of various advanced malignancies (2). The mechanism of action is inhibiting the ligand that leads to T-cell activation and inducing attack on the malignant cells (2, 3). These ligands include cytotoxic T-lymphocyte antigen-4 (CTLA-4), programmed death protein 1 (PD-1), and programmed death ligand-1 (PD-L1) (3). PD-1 inhibitors, pembrolizumab and nivolumab, have been approved by the Food and Drug Administration for treating unresectable or metastatic melanoma, Hodgkin lymphoma, non-small-cell lung cancer, head and neck squamous cell carcinoma, and renal cell carcinoma, and were recently applied on patients with ovarian cancer showing favorable results (4–6).

However, when upregulating the immune system, immune checkpoint inhibitors are accompanied with some unforeseen autoimmune-like side effects (1–3, 7). The most commonly reported ophthalmic manifestations are uveitis and dry eye, and others include inflammatory orbitopathy, keratitis, optic neuropathy, serous retinal detachment, extraocular muscle myopathy, atypical chorioretinal lesions, immune retinopathy, and neuroretinitis (3). Topical or periocular corticosteroids are used to manage the aforementioned inflammatory side effects, but systemic corticosteroids and cessation of checkpoint inhibitor therapy are required in refractory cases (8, 9).

Herein, we present an interesting case developing rare ocular immune-mediated adverse events of retinopathy other than uveitis, which are potentially associated with the use of the PD-1 inhibitors pembrolizumab and nivolumab for metastatic recurrent ovarian cancer.

## Case presentation

**Figure 1:**
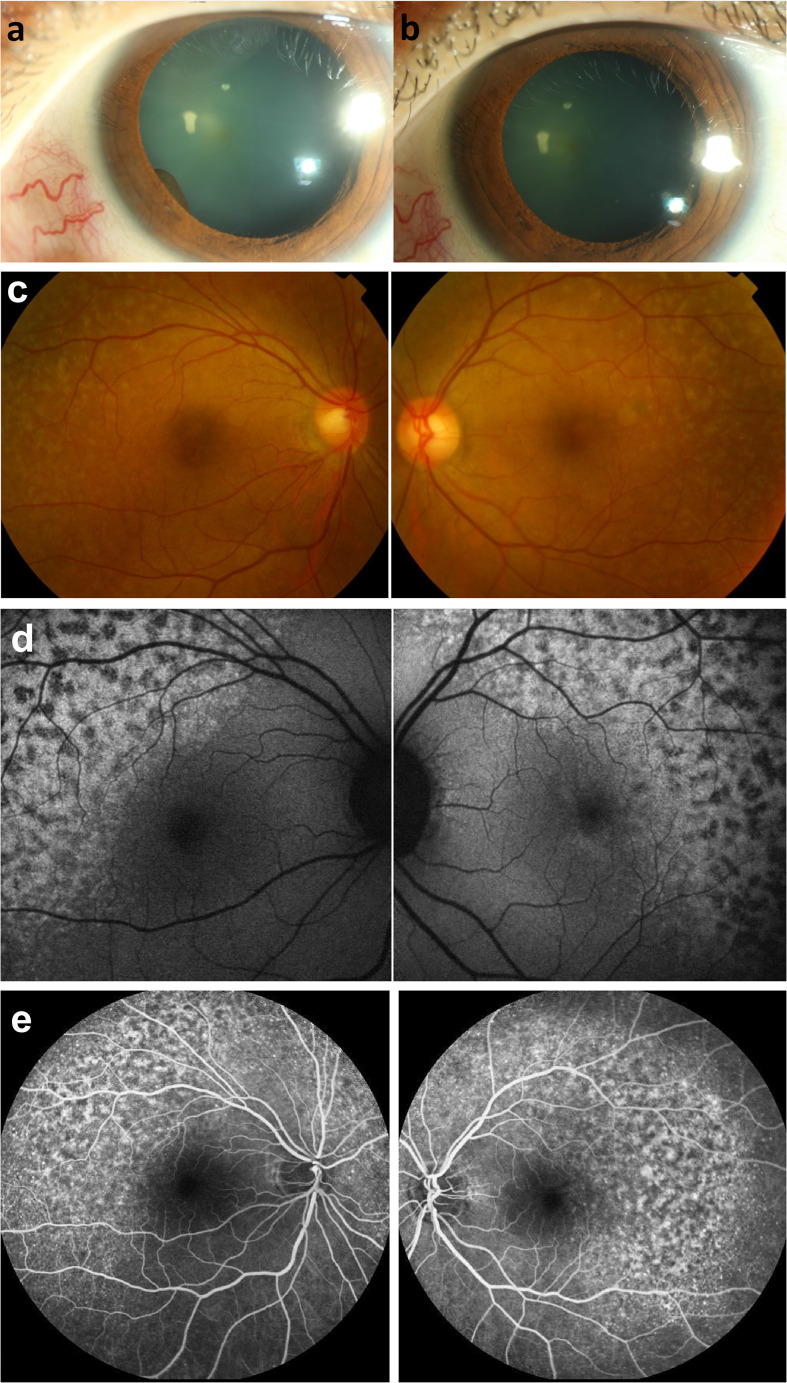
Photograpgh of the anterior eye. **(A)** an iris cyst was found 1 month after receiving immunotherapy with pembrolizumab. **(B)** resolution of anterior chamber cells and iris cysts after topical prednisolone and cycloplegics for 2 weeks. Fundus photography of fundus lesions in the bilateral eyes. **(C)** chorioretinal lesions with superotemporal macular mottling can be seen. Fundus autofluorescence (FAF) of fundus lesions in the bilateral eyes. **(D)** chorioretinal lesions show more prominent fundus changes with leopard appearance. Fluorescein angiographic features of the lesions in the bilateral eyes following the treatment with pembrolizumab. **(E)** late-phase fluorescein angiograph revealed symmetric strong staining with mild leakage over the lesions.

**Figure 2:**
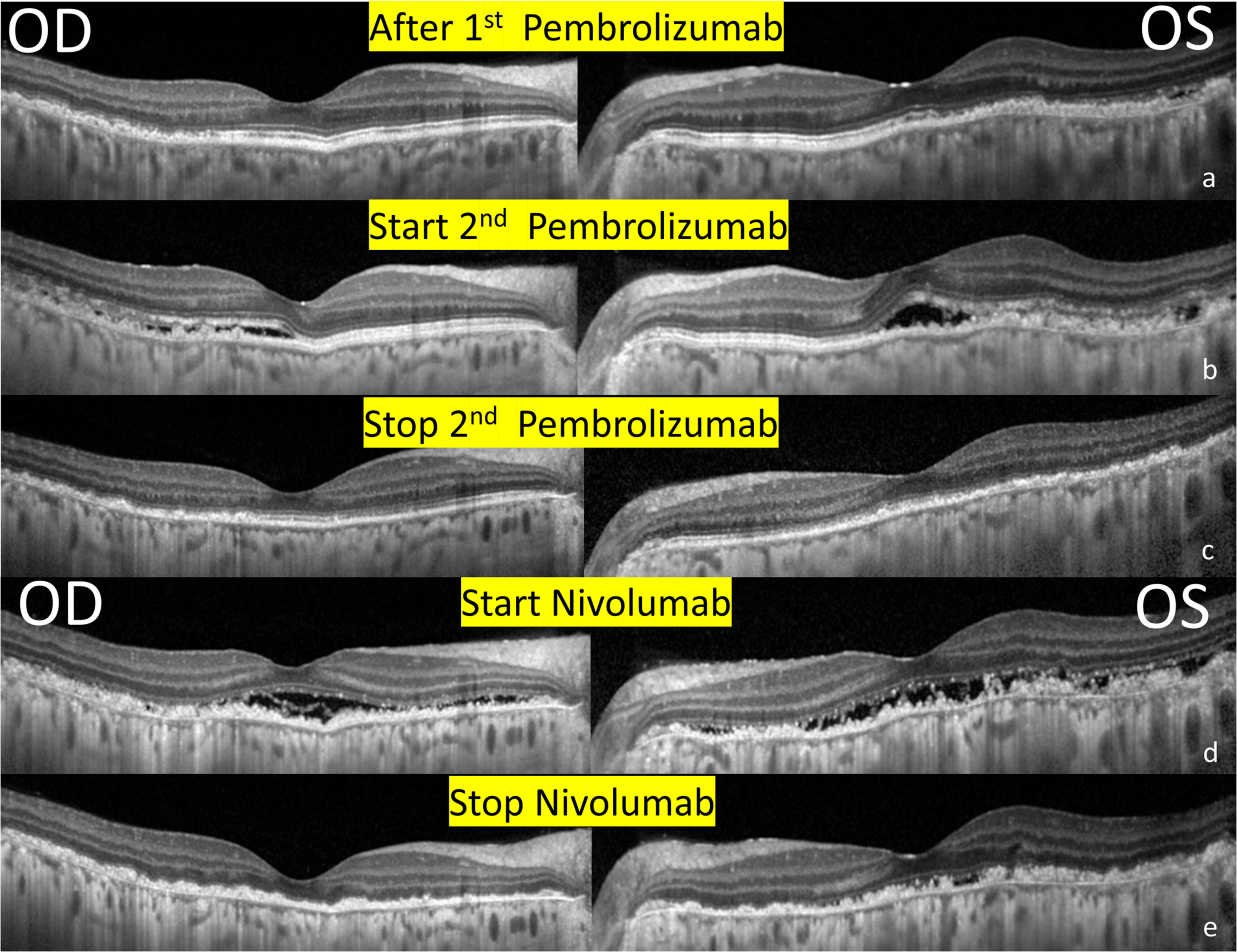
Serial changes in optical coherence tomography of the bilateral eyes. **(A)** 1 month after receiving immunotherapy with pembrolizumab. Note the RPE and outer retinal destruction over the heterogenous changes of the superior and temporal region. **(B)** 1 week after receiving 2^nd^ Pembrolizumab. Multiple subretinal fluids with RPE and outer retinal destruction involving the fovea developed. **(C)** After the discontinuation of pembrolizumab. A resolution of SRF but extensive RPE and outer retina destruction remained the same. **(D)** After receiving immunotherapy with nivolumab. New subretinal fluids with more prominent RPE and outer retinal destruction involving the fovea. **(E)** After the discontinuation of Nivolumab. A resolution of SRF and sustained extensive RPE and outer retina destruction.

A 37-year-old woman with recurrent metastatic ovarian cancer, who had undergone surgery, chemotherapy, and radiotherapy, presented to our clinic with blurred vision and photophobia in both the eyes 1 month after receiving immunotherapy with pembrolizumab (100mg triweekly). The Landolt C vision test revealed best-corrected visual acuity (BCVA) of 0.4 in the right eye and 0.2 in the left eye. Ocular findings were bilateral fine keratic precipitates (KPs), cells in the anterior chamber, iris cysts (**Figure 1A**), and yellowish flecks forming a reticulated leopard-spot pattern over superotemporal macula (**Figure 1C**). Corresponding fundus autofluorescence (FAF) showed more prominent fundus changes with patchy areas of hypoautofluorescence surrounded by zones of increased autofluorescence in a leopard spot pattern (**Figure 1D**). Optical coherence tomography (OCT) indicated disruptions and irregularities of RPE (RPE) and ellipsoid zone (EZ) over the heterogeneous changes on fundus exam, which could explain the RPE window defects on FAF; subretinal fluid (SRF) was also observed (**Figure 2A** **)**. The uveitis survey (complete blood count (CBC), ESR, CRP, treponemal and nontreponemal assays for syphilis, HLA-B27, ANA/Anti-phospholipid Ab/ANCA, CMV/VZV/HSV antibody, and chest X-ray) revealed no remarkable findings.

Her symptoms subsided after the application of topical prednisolone and cycloplegics for 2 weeks, with resolution of the anterior chamber cells and iris cysts (**Figure 1B**) (BCVA: 0.9/0.7). The immunotherapy was continued further for 4 months (pembrolizumab 100mg triweekly for 6 cycles) with satisfactory clinical effects. However, the fundus appearance persisted after cessation of the immunotherapy.

However, metastasis recurred after 1 year, and pembrolizumab was administered again (100mg triweekly). One week after receiving pembrolizumab, she complained of redness and blurred vision in both the eyes. BCVA was 0.5/0.4, and ocular examinations revealed bilateral fine KPs and anterior chamber cells. SRF, and disruptions and irregularities of RPE and EZ were observed (**Figure 2B**), especially over the inferior retina. Fluorescein angiography demonstrated strong staining and mild leakage over the lesions (**Figure 1E**). With oral and topical steroids, the anterior chamber reaction subsided, but there were no obvious effects on SRF. Pembrolizumab had been continued for three cycles, but the metastasis was not well-controlled. After the discontinuation of pembrolizumab, macular OCT revealed a resolution of SRF, but extensive RPE and outer retina destruction persisted, without obvious vision improvement (**Figure 2C**).

One month later, immunotherapy with nivolumab (160mg biweekly) was performed. The patient complained of darker vision in both the eyes, and a BCVA of 0.3/0.1 was detected. Ocular examinations revealed mild anterior chamber cells and exudative SRF over the macula (**Figure 2D**). The patient was averse to the use of oral and topical steroids because of the absence of apparent effects. Nivolumab was discontinued after three cycles, with resolution of the anterior chamber reaction and SRF (**Figure 2E**) and no further regain in the visual acuity.

## Discussion and conclusion

Owing to the enhancement of the immune system and activation of the T cells to generate an antitumor response, immune checkpoint inhibitors have emerged as a method for the management of previously incurable malignancies. The primary targets for checkpoint inhibition include CTLA-4 and PD-1 ligands, which reside directly on the T-cell membrane and promote T-cell downregulation. Once they are blocked, T cells are activated (3, 10). Immune activity is also stimulated after the blockage of PD-L1 residing on the tumor cells (3). Pembrolizumab and nivolumab are the PD-1 inhibitors, which were previously indicated for metastatic melanoma and have recently been applied widely to other unresectable or metastatic solid tumors with certain genetic anomalies (5, 9). However, studies on gynecologic cancers are limited compared to studies on cancers of other sites (11). In this case, as the recurrent metastatic ovarian cancer was refractory to surgery, chemotherapy, and radiotherapy, the gynecologist provided immune therapy to the patient, which resulted in irreversible RPE changes and ellipsoid-band disruption, even with macular involvement.

Up to 50% of patients, receiving immunotherapy reported systemic complications, such as peripheral edema, fatigue, pruritus, skin rash, elevation in liver enzyme levels, and other endocrine, gastrointestinal, and hematological events (12). In contrast, ocular side effects were reported to be rare. Intraocular inflammation (uveitis) following the treatment with pembrolizumab, nivolumab, or cemiplimab is a rare but clinically important event, described in approximately 1% of treated patients. It mostly manifests as bilateral anterior uveitis and may persist or recur even after termination of the immune therapy (12, 13). Other reported ocular side effects include conjunctivitis, episcleritis, Graves-like orbitopathy, and papillitis (7, 14). Chorioretinal lesions following the administration of pembrolizumab was only elucidated in case reports, presenting as shallow choroidal effusion with bilateral focal exudative retinal detachment, posterior uveitis (6), multifocal choroiditis resulting in chorioretinal scars, and pigment clumps over the peripheral fundus (15), which were different from the presentation and location in our patient. Novel findings of the macular involvement with the presentation of posterior uveitis, serous retinal detachment and subretinal fluid in association with the PD-1 inhibitors were announced recently in rare cases (13, 16).

Although relatively uncommon, ocular side effects from systemically administered immunotherapy can be serious, incapacitating, and irreversible. A conceivable hypothesis suggests that the “unleashed” autoimmunity is triggered by checkpoint inhibitors (15). In 2017, the Society for Immunotherapy of Cancer toxicity management working group announced recommendations by consensus for managing toxicities associated with immune checkpoint inhibitors (17). The group recommended topical or periocular steroids with temporarily holding PD-1 inhibitors for grade 3 adverse events (anterior uveitis with 3+ or greater cells; intermediate, posterior, and panuveitis) while permanently discontinuing PD-1 inhibitors for grade 4 uveitis (events resulting in visual acuity 20/200 or worse). However, standard treatment strategies have not been well established for ocular side effects other than local and systemic steroids in addition to holding PD-1 inhibitors (6). Previously reported cases showed regained vision and complete recovery of symptoms after cessation of the immunotherapy (6). On the other hand, fundus depigmentation with progressive choroidal thinning and absence of ocular inflammations (18), and fundus depigmentation with thickened (or normal) choroid after choroidal choroiditis (19, 20) had both been mentioned in previous case reports. Although no specific changes of choroidal thickness were detected in our case, Indocyanine green (ICG) angiography may help evaluating eventual choroidal involvement in certain cases. Moreover, recent studies also proposed that the implicit times of the flicker electroretinogram (ERG) were significantly prolonged and the amplitudes were significantly reduced at the initial stages (21). While not performed in our case, flicker ERG could play a helpful tool in monitoring the recovery of retinal function before and after the steroid treatment. In our case, the patient received pembrolizumab twice and nivolumab once for metastatic ovarian cancer and exhibited reversible anterior uveitis, iris cysts, exudative SRF, and unforeseen irreversible RPE changes and ellipsoid-band disruption in both the eyes. Anterior uveitis and iris cysts were reversible after the application of topical steroids and cycloplegics while SRF was refractory to systemic high-dose steroids but spontaneously resolved after the immunotherapy was ceased. However, even after the resolution of SRF, RPE and ellipsoid-band disruption were progressive and irreversible, and the vision remained blurred due to macular involvement.

Owing to the possible irreversible ocular complications, the initial thorough baseline ocular examinations and close follow-up of patients before and during the treatment with immune checkpoint inhibitors should be prudently considered. Once ocular toxicity occurs, the decision to cease or continue the therapy must be individualized. According to the study involving patients newly diagnosed with ophthalmic immune-related adverse effects during 2013 to 2017 from American Academy of Ophthalmology’s Intelligent Research in Sight (IRIS^®^) Registry, elevated rates of ophthalmic immune-related adverse effects in patients under immune checkpoint inhibitors were reported when compared with ocular complication rates in the entire registry population (22). Consequently, early multidisciplinary collaboration of oncologists and ophthalmologists is important. In patients whose vision is threatened, and simultaneously, no effective alternative for systemic treatment exists, the benefits of continuing the specific immunotherapy must be weighed against the risks and consequences of ocular toxicity.

In conclusion, this case report demonstrates that pembrolizumab- and nivolumab-related ocular side effects can include bilateral anterior uveitis, iris cysts, exudative SRF, RPE and ellipsoid-band disruption involving the fovea. Oncologists and ophthalmologists should pay attention to the possible ocular changes during the treatment course. The benefits of continuing the immunotherapy must be weighed against the risks and consequences of ocular toxicities.

## Data availability statement

All data generated or analyzed during this study are included in this article, further inquiries can be directed to the corresponding author.

## Ethics statement

The studies involving human participants were reviewed and approved by Ethics committee approval was obtained from Chang Gung Medical Foundation Institutional Review Board (No. 202000018B0). The patients/participants provided their written informed consent to participate in this study. Written informed consent was obtained from the next of kin for the publication of any potentially identifiable images or data included in this article. This study waives informed consents according to our IRB regulation and the patient had passed away in May 2019. The consent for publication was obtained from her husband.

## Author contributions

Conceptualization: W-YC. Data curation: W-HC and W-YC. Writing – original draft: W-HC. Writing – review & editing: W-YC. All authors contributed to the article and approved the submitted version.
